# Diel CO_2_ cycles reduce severity of behavioural abnormalities in coral reef fish under ocean acidification

**DOI:** 10.1038/s41598-017-10378-y

**Published:** 2017-08-31

**Authors:** Michael D. Jarrold, Craig Humphrey, Mark I. McCormick, Philip L. Munday

**Affiliations:** 1 College of Science and Engineering, James Cook University, Townsville, QLD 4811 Australia; 2 ARC Centre of Excellence for Coral Reef Studies, James Cook University, Townsville, QLD 4811 Australia; 3 National Sea Simulator, Australian Institute of Marine Science, PMB 3, Townsville, Queensland 4810 Australia

## Abstract

Elevated CO_2_ levels associated with ocean acidification (OA) have been shown to alter behavioural responses in coral reef fishes. However, all studies to date have used stable *p*CO_2_ treatments, not considering the substantial diel *p*CO_2_ variation that occurs in shallow reef habitats. Here, we reared juvenile damselfish, *Acanthochromis polyacanthus*, and clownfish, *Amphiprion percula*, at stable and diel cycling *p*CO_2_ treatments in two experiments. As expected, absolute lateralization of *A. polyacanthus* and response to predator cue of *Am. percula* were negatively affected in fish reared at stable, elevated *p*CO_2_ in both experiments. However, diel *p*CO_2_ fluctuations reduced the negative effects of OA on behaviour. Importantly, in experiment two, behavioural abnormalities that were present in fish reared at stable 750 µatm CO_2_ were largely absent in fish reared at 750 ± 300 µatm CO_2_. Overall, we show that diel *p*CO_2_ cycles can substantially reduce the severity of behavioural abnormalities caused by elevated CO_2_. Thus, past studies may have over-estimated the impacts of OA on the behavioural performance of coral reef fishes. Furthermore, our results suggest that diel *p*CO_2_ cycles will delay the onset of behavioural abnormalities in natural populations.

## Introduction

Increasing atmospheric CO_2_ levels are expected to cause a reduction of ocean surface water pH by 0.3–0.4 of a unit by the year 2100, a process commonly referred to as ocean acidification (OA)^1^. Ocean acidification projections are based on open ocean environments that are relatively stable over time^1^. In contrast, coastal and shallow water habitats can experience substantial natural fluctuations in *p*CO_2_ on a variety of temporal scales^2, 3^. These fluctuations are driven by a range of biological and physical processes^4^ and in some instances their magnitude can exceed mean CO_2_ levels projected to occur over the next century^2, 3^. Furthermore, natural *p*CO_2_ fluctuations are expected to increase in size throughout the century, as increased CO_2_ uptake by the oceans leads to reduced seawater buffering capacity^5, 6^. Consequently, as mean oceanic *p*CO_2_ levels rise, shallow water marine organisms will be exposed to higher *p*CO_2_ levels for longer periods of time in addition to experiencing a greater range of *p*CO_2_ levels.

Our current understanding of how natural *p*CO_2_ fluctuations will interact with rising mean oceanic *p*CO_2_ levels to affect the performance of shallow water marine organisms under future OA is limited. This is because most OA experiments have used stable *p*CO_2_ levels consistent with open ocean projections, instead of *p*CO_2_ levels naturally relevant to the study organism^7, 8^. While such experiments have demonstrated a range of impacts on traits across various taxa^9–11^, their ecological relevance is uncertain. Indeed, a handful of studies have shown that natural *p*CO_2_ fluctuations can significantly modify the biological responses of shallow water marine organisms to OA^12–19^. Consequently, there has been a call for experiments on shallow water marine organisms that include *p*CO_2_ treatments representative of their natural habitats^7, 8, 20, 21^. Results from such experiments will be vital for improving predictions of when the negative effects caused by elevated *p*CO_2_ will become evident in natural populations^22^.

Some of the most notable effects of stable, elevated *p*CO_2_ levels have been observed in coral reef fishes. Specifically, exposure to *p*CO_2_ levels between 700–1000 µatm have been shown to impair a range of sensory systems and alter ecologically important behaviours^10, 23, 24^. Alterations include impaired anti-predator responses^25–29^, loss of lateralization^30, 31^, loss of learning^32, 33^ and increased activity/boldness^27^. Such behavioural abnormalities are expected to have significant ecological consequences for fish populations. For example, as a consequence of exhibiting riskier behaviour, predation-related mortality was significantly higher when settlement stage damselfish were exposed to elevated *p*CO_2_ in the laboratory and released into their native habitat, inferring that recruitment and population sustainability will be threatened by projected future CO_2_ levels in the ocean^27^. Furthermore, the impacts that behavioural abnormalities have on predator-prey dynamics^26, 34^ and competitive interactions^35^ will likely cause shifts in community structure with unknown consequences for ecosystem functioning.

Coral reefs are highly dynamic shallow water habitats that experience diel cycles in *p*CO_2_. These daily CO_2_ cycles are driven by the processes of photosynthesis/respiration and calcification/dissolution over a day-night cycle, but are also influenced by physical controls such as water flow and residence time^36–38^. In shallow reef areas, diel variation in *p*CO_2_ can range anywhere from ±50 to 600 µatm around the mean^38–41^. Although the *p*CO_2_ of coral reef waters is not in perfect equilibrium with the atmosphere over a daily timescale, the carbonate system is still heavily influenced by flushing with offshore waters and thus the mean *p*CO_2_ of reef waters will rise in line with rising atmospheric CO_2_^36^. To our knowledge, only three studies (all on calcifying corals) have explicitly considered diel *p*CO_2_ variation when investigating the potential impacts of OA on coral reef organisms. Importantly, they found that the negative impacts of OA on growth and calcification were buffered by the presence of a diel cycling *p*CO_2_ regime^12, 13, 42^. The behavioural alterations that have been observed in coral reef fishes are likely to be sensitive to the interactive effects of diel *p*CO_2_ cycles and rising mean *p*CO_2_ levels for two reasons. Firstly, previous work has shown that it takes between 24–96 h of exposure to stable elevated *p*CO_2_ levels for behavioural abnormalities to manifest, with shorter onset times at higher *p*CO_2_ levels^27^. Secondly, the negative effects of elevated *p*CO_2_ on behavioural responses are concentration-dependent^27, 28, 31^. Consequently, diel *p*CO_2_ cycles could reduce the severity of behavioural abnormalities, or prevent them from manifesting, by providing fish with a recovery period, especially if *p*CO_2_ levels drop below the onset threshold (600–700 µatm). Alternatively, experiencing higher maximum *p*CO_2_ levels daily may lead to more severe behavioural abnormalities.

To determine how diel *p*CO_2_ cycles affect the behavioural responses of coral reef fishes under OA, we reared juvenile damselfish, *Acanthochromis polyacanthus* (Bleeker, 1855), and clownfish, *Amphiprion percula* (Lacepède, 1802), under a series of stable and diel cycling *p*CO_2_ treatments in two different experiments. The aim of the first experiment was to determine if the magnitude of diel *p*CO_2_ cycles affects the behavioural performance of coral reef fishes under OA. The aim of the second experiment was to determine if the presence of diel *p*CO_2_ cycles affects the mean CO_2_ level at which behavioural abnormalities occur (i.e. the onset of behavioural abnormalities). Specifically, in experiment one, the behaviour of fish reared at two stable CO_2_ levels (480 and 1000 µatm) was compared with the behaviour of fish reared in two cycling CO_2_ treatments of different magnitude (1000 ± 300 and 1000 ± 500 µatm). Therefore, this experiment enabled us to test if the magnitude of diel *p*CO_2_ fluctuations affected the behaviour of fish under OA. In experiment two, the behaviour of fish reared at three stable CO_2_ levels (460, 750 and 1000 µatm) was compared with the behaviour of fish reared in diel cycling CO_2_ treatments at two different mean CO_2_ levels (750 ± 300 and 1000 ± 300 µatm). Therefore, this experiment enabled us to test if the effect of diel *p*CO_2_ cycles was dependent on the mean CO_2_ level experienced by the fish. In both experiments, we measured behavioural lateralization in *A. polyacanthus* and the response to a predator cue by *Am. percula*. These traits were chosen for each species as previous studies have demonstrated clear negative impacts of exposure to stable, elevated *p*CO_2_ conditions^25, 27, 31, 43^. It was predicted that diel *p*CO_2_ fluctuations could reduce the overall severity and delay the onset of behavioural abnormalities under OA conditions.

## Results

### Experiment one

**Figure 1:**
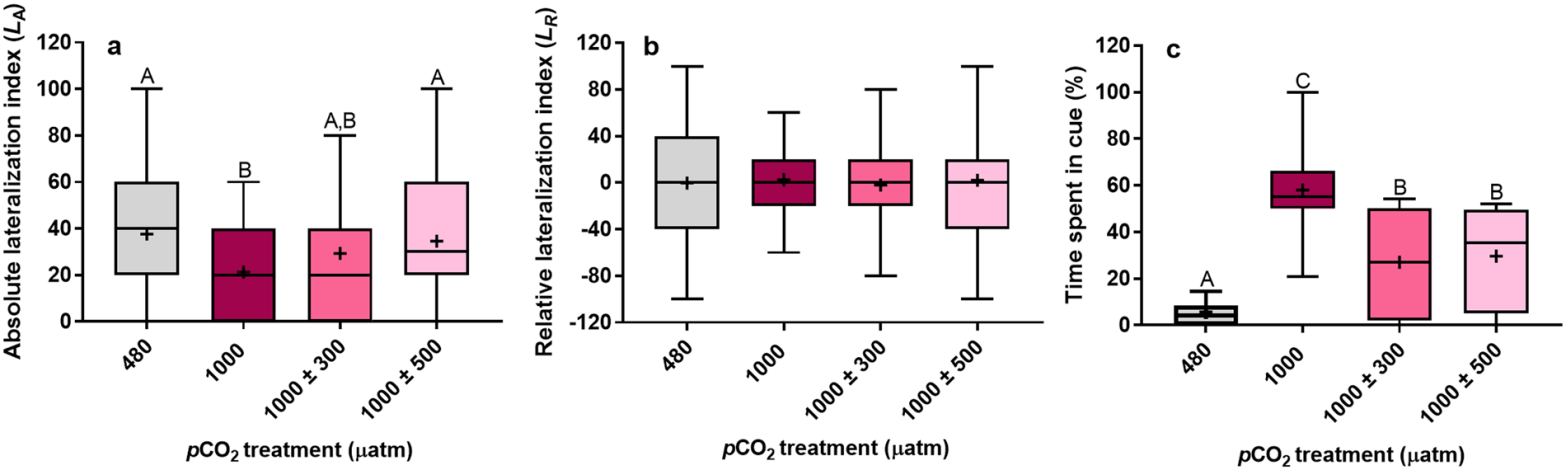
Effects of stable vs diel cycling elevated *p*CO_2_ on behavioural responses in experiment one. (**a**) Absolute lateralization and (**b**) relative lateralization in juvenile *Acanthochromis polyacanthus* (n = 60 per treatment) were determined using a two-way T-maze. (**c**) Response to predator cue of juvenile *Amphiprion percula* (n = 16 per treatment) was determined using a two-choice flume. Different letters represent significant differences between treatments (Tukey, *P* < 0.05). Boxplots are sized according to the 25th and 75th quartiles, where the line identifies the median and the whiskers indicate the minimum and maximum values. +signs represent means.

Absolute lateralization (*L*_*A*_) was significantly influenced by CO_2_ treatment (Fig. 1a, χ^2^ = 15.75, df = 3, *P* = 0.001). As expected, juveniles reared under stable, elevated *p*CO_2_ were less lateralized compared to those reared at control levels (*P* = 0.001). However, diel *p*CO_2_ cycles significantly increased how lateralized juvenile *A. polyacanthus* were at 1000 µatm. *L*_*A*_ of juveniles reared under small fluctuations (±300 µatm) was intermediate, but not significantly different, to those reared at control and stable, elevated *p*CO_2_ (min. *P* = 0.214). *L*_A_ of juveniles reared under large fluctuations (±500 µatm) was fully restored to control levels being significantly greater than those reared at stable, elevated *p*CO_2_ (*P* = 0.01). Mean relative lateralization (*L*_R_) in juvenile *A. polyacanthus* was unaffected by CO_2_ treatment (Fig. 1b, χ^2^ = 0.52, df = 3, *P* = 0.914). Furthermore, no group exhibited a preference for left or right turning (Fig. S1, max. χ^2^ = 0.84, *P* = 0.358). Juveniles reared under stable, elevated *p*CO_2_ tended to have a narrower *L*_R_ distribution compared to the other treatments (Fig. S3), although these differences were not significant (Max. KS = 0.15, *P* = 0.510).

Mean percentage time that juvenile *Am. percula* spent in predator cue water was significantly affected by CO_2_ treatment (Fig. 1c, χ^2^ = 51.45, df = 3, *P* < 0.001). As expected, juveniles reared at stable, elevated *p*CO_2_ spent a greater amount of time in predator cue water compared to those reared at control levels (*P* < 0.001). However, diel *p*CO_2_ cycles significantly reduced the amount of time that juvenile *Am. percula* spent in predator cue water at 1000 µatm. Juveniles reared under both small (±300 µatm) and large (±500 µatm) fluctuations demonstrated partial restoration of antipredator behaviour spending an amount of time in predator cue water which was intermediate, and significantly different, to juveniles reared at control and stable, elevated *p*CO_2_ (max. *P* < 0.001).

### Experiment two

**Figure 2:**
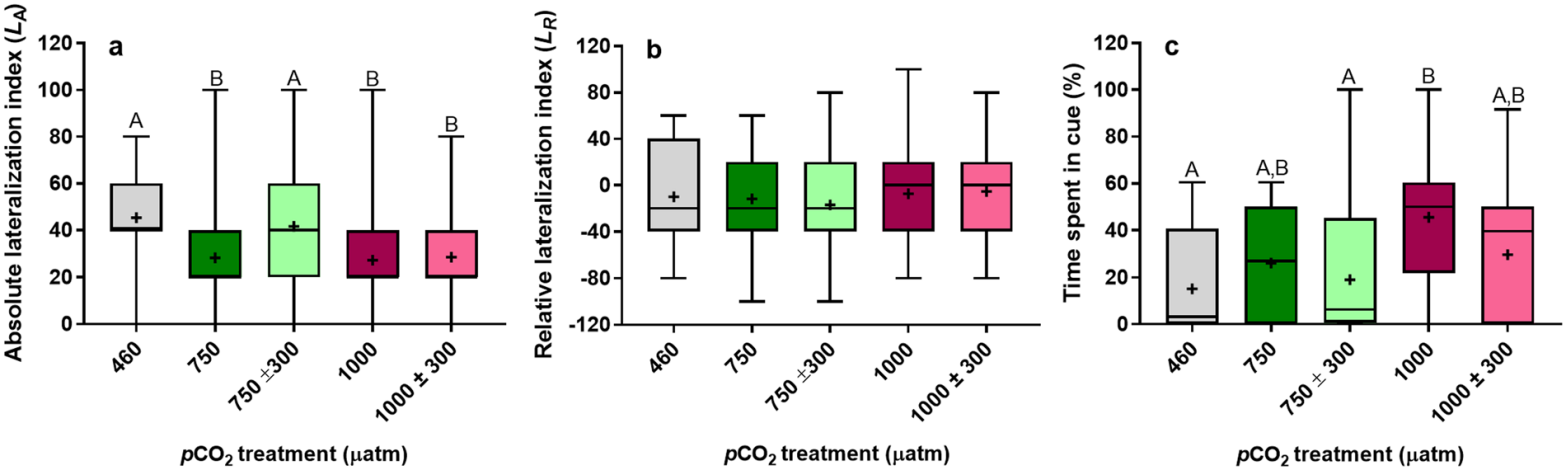
Effects of stable vs diel cycling elevated *p*CO_2_ on behavioural responses in experiment two. (**a**) Absolute lateralization and (**b**) Relative lateralization in juvenile *Acanthochromis polyacanthus* (n = 60 per treatment) were determined using a two-way T-maze. (**c**) Response to predator cue of juvenile *Amphiprion percula* (n = 24 per treatment) was determined using a two-choice flume. Different letters represent significant differences between treatments (Tukey, *P* < 0.05). Boxplots are sized according to the 25th and 75th quartiles, where the line identifies the median and the whiskers indicate the minimum and maximum values. +signs represent means.

As was observed in experiment one, mean *L*_*A*_ was significantly affected by CO_2_ treatment (Fig. 2a, χ^2^ = 75.25, df = 4, *P* < 0.001), with juveniles reared under stable, elevated *p*CO_2_ (750 and 1000 µatm) being less lateralized compared to those reared at control levels (max. *P* < 0.001). Diel *p*CO_2_ cycles did not affect how lateralized juvenile *A. polyacanthus* were at mean *p*CO_2_ level of 1000 µatm (*P* = 0.986). In contrast, diel *p*CO_2_ cycles fully restored lateralization in juvenile*s* reared at a mean CO_2_ of 750 µatm, being similar to those reared at control levels (*P* = 0.710) and significantly greater than both the stable, elevated CO_2_ treatments (max. *P* < 0.001). Also, as observed in experiment one, mean *L*_R_ in juvenile *A. polyacanthus* was unaffected by CO_2_ treatment (Fig, 2b, χ^2^ = 4.86, df = 4, *P* = 0.302), and no group exhibited a preference for left or right turning (Fig. S2, max. χ^2^ = 3.43, *P* = 0.064). However, there were more individuals which were less lateralized in the 750, 1000 and 1000 ± 300 µatm CO_2_ treatments (Fig. S2).

Similar to experiment one, CO_2_ treatment significantly affected the mean percentage time that juvenile *Am. percula* spent in predator cue water (Fig. 2c, χ^2^ = 15.95, df = 4, *P* = 0.003). As expected, juveniles reared at 1000 µatm CO_2_ spent a greater amount of time in predator cue water compared to those reared at control levels (*P* = 0.004). The percentage time juveniles reared at 750 µatm CO_2_ spent in predator cue water was intermediate, but not significantly different, to those reared at control and 1000 µatm *p*CO_2_ (min. *P* = 0.194). Diel *p*CO_2_ cycles influenced the predator cue response of juvenile *Am. percula* at mean *p*CO_2_ levels of 750 and 1000 µatm. Juveniles reared at 750 ± 300 µatm CO_2_ spent a percentage of time in predator cue water which was more similar to those reared at control levels compared to those reared at 750 µatm CO_2_. Finally, juveniles reared at 1000 ± 300 µatm CO_2_ demonstrated partial restoration of antipredator behaviour, with juveniles spending a percentage of time in predator cue water which was intermediate to those reared at 460 and 1000 µatm CO_2_ (min. *P* = 0.309).

## Discussion

This study demonstrates for the first time that diel *p*CO_2_ cycles can significantly modify the behavioural responses of fishes under OA. The negative impacts of elevated CO_2_ on coral reef fish behaviour have been well documented and are expected to have significant ecological consequences for reef fish populations through effects on recruitment, predator-prey interactions, competition and habitat preference^10, 23, 24^. However, all studies to date have exposed fish to stable levels of elevated CO_2_, not considering the natural diel *p*CO_2_ cycles that occur on coral reefs. Here we show that the severity of two behavioural abnormalities commonly observed under elevated CO_2_ are reduced when fish experience a diel cycling *p*CO_2_ regime. The extent of reduction was influenced by both the magnitude of fluctuation and mean *p*CO_2_ level experienced, as well as the behavioural trait. Overall, our results indicate that previous studies have probably over-estimated the behavioural impacts of OA on coral reef fishes once they have settled to reef habitats where diel CO_2_ cycles are prevalent.

Previous research using stable *p*CO_2_ treatments has found that behavioural abnormalities start to manifest in coral reef fish between 600–700 µatm. Our results indicate that diel *p*CO_2_ cycles will delay the onset of behavioural abnormalities. In experiment two, we show that behavioural abnormalities present in fish reared at a stable level of 750 µatm CO_2_ were absent in fish reared at 750 ± 300 µatm CO_2_. However, in both experiments, although less severe, behavioural abnormalities were still present in the fluctuating 1000 µatm CO_2_ treatments. Thus, it appears that mean oceanic *p*CO_2_ levels closer to 1000 µatm will need to be reached before behavioural abnormalities could manifest in natural populations of reef fishes. Furthermore, we observed full restoration of behavioural lateralization in juvenile *A. polyacanthus* reared under 1000 ± 500 µatm CO_2_, inferring that some behavioural abnormalities may not manifest at all for populations living in habitats with large CO_2_ fluctuations, such as shallow reef flats and closed lagoons, even when average oceanic conditions reach 1000 µatm CO_2_. The observation that diel *p*CO_2_ variation can reduce and/or delay the onset of behavioural abnormalities in juvenile coral reef fish under OA is particularly important given the ecological consequences of behavioural abnormalities and past research that has shown a limited capacity for acclimation of behavioural traits to stable, elevated *p*CO_2_^31, 44^. However, it is important to mention that behavioural abnormalities are still likely to occur in the pelagic larval phase of coral reef fish as they occupy a more stable CO_2_ environment in the open ocean. Consequently, population replenishment and sustainability of reef fish populations could still be threatened by near-future OA due to impaired behaviour in the larval phase^27, 45, 46^, even if behavioural effects are less severe in juveniles that have already settled to reef habitats. Finally, in experiment one, and to a lesser extent in experiment two, we observed more individual variation in predator cue responses of *Am. percula* at 1000 µatm CO_2_ if fish were reared under cycling conditions. This level of individual variation has previously been observed only at a mean stable CO_2_ of 700 µatm^27^. Thus, in addition to potentially providing more time for reef fish populations to adapt to future OA conditions, by delaying the onset of behavioural abnormalities, diel *p*CO_2_ cycles may also increase the adaptive potential of fish populations at higher CO_2_ levels by increasing the range of individual variation upon which selection can act.

The underlying mechanism of behavioural abnormalities in fish under OA conditions is linked to the effects of acid-base regulation on the function of type A γ-aminobutyric acid (GABA_A_) neurotransmitter receptors^23, 43^. GABA_A_ receptors are gated ion channels with specific conductance for HCO_3_^−^ and Cl^−^. Under elevated *p*CO_2_ fish increase intracellular and extracellular HCO_3_^−^ concentrations to prevent plasma and tissue acidosis^47–49^. In a recent study on *A. polyacanthus* this compensatory mechanism was shown to be sufficient to reverse the transmembrane gradients of HCO_3_^−^ in brain tissue, which could interfere with GABA_A_ receptor function and cause behavioural alterations^50^. For coral reef fish it appears that complete acid-base regulation in the brain under stable, elevated *p*CO_2_ levels may take between 24–96 h, as this is the exposure period required before behavioural abnormalities manifest^27^. Our results suggest that for fish reared under diel *p*CO_2_ cycles, exposure to lower CO_2_ levels for several hours each day is sufficient to prevent the physiological changes that would normally occur at a stable, high CO_2_. Extracellular and intracellular pH regulation take place at different rates, occurring more quickly in the former. For example, in gulf toadfish (*Opsanus beta*) exposed to 1900 μatm CO_2_, complete blood pH compensation was achieved after 2 h, whereas muscle intracellular pH was not adjusted until after 24h^49^, see also^51^. Thus, based on the onset times under stable, elevated *p*CO_2_ (24–96 h), it would appear that behavioural abnormalities do not manifest in coral reef fish until brain intracellular pH compensation is complete, although further testing is required. This could explain why diel *p*CO_2_ cycles alleviated the negative impacts of OA. Although no data is available, we assume coral reef fish would achieve pH compensation as fast, or faster, then toadfish in the example above, due to their higher metabolic rates and more active lifestyle. Therefore, we hypothesise that fish reared under diel *p*CO_2_ cycles were able to track disturbances in extracellular pH, but weren’t exposed to higher *p*CO_2_ levels long enough for full brain intracellular pH compensation to occur.

It has been suggested that behavioural abnormalities may also be influenced by alterations in gene expression related to ion regulation^32, 52^. Ion-regulation in blood and tissues is under circadian control in fishes^53, 54^. In a recent study on *A. polyacanthus*, variation in behavioural tolerance to stable, elevated *p*CO_2_ (754 µatm) was linked to the differential expression of genes related to circadian rhythm control^55^. For example, offspring of CO_2_ sensitive parents (i.e., those that exhibited behavioural abnormalities) upregulated the enzyme that catalyses the final reaction in the synthesis of melatonin, a key regulator of the circadian rhythm, which plays an important role in controlling ion-regulation^56^. This indicates that CO_2_ sensitive individuals might display more pronounced acid-base compensation if exposed to a sustained elevation of CO_2_ due to a stronger influence of circadian rhythm control, leading to larger changes of the neuronal ion gradients that determine GABA_A_ receptor function. Our observations that diel *p*CO_2_ cycles can alleviate the negative behavioural effects of OA suggests that fish were displaying normal, or less, circadian control over acid-base regulation and thus did not respond so strongly to internal pH changes caused by elevated CO_2_ therefore avoiding altered brain ion gradients. Consequently, it appears that internal circadian rhythm control of acid-base regulation in coral reef fish is disrupted under stable, elevated *p*CO_2_, indicating that this process may be linked to the natural diel *p*CO_2_ cycles occurring in shallow reef habitats.

In this study we repeated the control, stable 1000 µatm CO_2_ and 1000 ± 300 µatm CO_2_ treatments in two different experiments. Although we observed similar responses to predator cue in *Am. percula* in both experiments, there were some differences in the effects of CO_2_ cycles on behavioural lateralization. In experiment one behavioural lateralization was partially restored in juvenile *A. polyacanthus* reared at 1000 ± 300 µatm CO_2_, whereas no restoration was observed in experiment two. The reason for the different results between experiments is unclear, but one possible reason is differences in the duration that the high CO_2_ peaks lasted. In experiment one the high peaks lasted approximately three hours, whereas in experiment two they lasted close to eight hours. Consequently, fish in experiment one had less time to adjust their acid-base status during the high peak, which may have resulted in them exhibiting less severe behavioural impairments. The reason we did not observe a similar difference between experiments for response to predator cue in juvenile *Am. percula* may be because the effect of elevated *p*CO_2_ on this trait was concentration dependent, as seen in experiment two. Due to logistical constraints, it was not possible to have duplicated experimental systems in experiment one. In contrast, experiment two had duplicate systems for each *p*CO_2_ treatment. As similar results were observed in each experiment we are confident that the pseudo-replication in experiment one did not affect the results. In general, the effects of stable, elevated *p*CO_2_ on lateralization and response to a predator cue observed in this study are consistent with previous work on the same species^25, 27, 29, 31^, with one exception. Previous studies have reported a clear attraction of *Am. percula* to a predator cue (>80% of time in predator cue water) at 1000 µatm CO_2_, whereas *Am. percula* in the current experiments exhibited neither attraction of avoidance of the predator cue (45–58% of time in predator cue water) at this CO_2_ level. The same observation was also reported in adult goldskinny wrasse, *Ctenolabrus rupestris* ^57^. Why fish in the current experiments exhibited a less dramatic change in antipredator behaviour at high CO_2_ compared with previous experiments is unknown, but could be related to some differences in protocol. In contrast to past studies that reset each fish to the starting position when the direction of the water sources was switched, fish were not disturbed during trials in this study. Another potential factor is the life-stage that was tested. Previous studies tested settlement stage larvae, whereas settled juveniles were used in this study. Age-specific responses to predator cues, as well as expression of odourant receptor genes, have been observed in other species of fish^58, 59^.

In this study, we show that a diel *p*CO_2_ cycle can substantially reduce the severity of behavioural abnormalities caused by elevated CO_2_ in coral reef fishes. In contrast, behavioural impairments were still present in a temperate shark species reared under elevated CO_2_ in a mesocosm that experienced diel CO_2_ variation, although there was no stable, elevated CO_2_ treatment to compare against^60^. A handful of other studies have also shown that daily *p*CO_2_ fluctuations can significantly modify the biological responses of shallow water marine organisms to OA^12–15, 17, 19^. This highlights the importance of considering natural *p*CO_2_ variability when trying to determine the response of shallow water marine organisms to OA. While our understanding of the magnitude and frequency of *p*CO_2_ fluctuations *in situ* is growing, many shallow water habitats remain under- or un-sampled^8^. Consequently, there is a need for more high resolution *in situ* studies that characterise natural CO_2_ variability both spatially and temporally. Such data will establish ecologically relevant *p*CO_2_ treatments to be used in laboratory experiments and allow us to better interpret results from past OA studies that have employed stable *p*CO_2_ levels^7, 61^. This will be critical for accurately assessing the likely effects of OA on shallow water marine organisms and which species and ecosystems may be at greatest risk.

## Materials and Methods

### Study species

*Acanthochromis polyacanthus* and *Amphiprion percula* are common throughout the Indo-Pacific region. Both species are demersal spawners, laying their eggs within small caves and crevices in the reef matrix. In *A. polyacanthus*, eggs hatch into small juveniles, with both parents providing care to the eggs and offspring for up to 45 d post-hatching^62^. In contrast, *Am. percula* has a relatively short larval phase of approximately 11 d before settling on the reef ^63^. Both species can be bred and reared in captivity with high success, which has led to their establishment as models for investigating the potential impacts of OA on coral reef fishes^25, 27, 31, 50, 55^.

### Brood-stock and general rearing protocol

Adult *A. polyacanthus* were collected using hand nets from the Bramble Reef area (site 1: 18°22′S, 146°40′E; site 2: 18°25′S, 146°40′E) of the Great Barrier Reef in July 2015. Fish were transported to an environmentally controlled aquarium research facility at James Cook University (JCU) (Townsville, Australia) where they were housed as breeding pairs in 60 L aquaria at temperature conditions matching the collection location. An existing brood-stock of *Am. percula* at JCU was used. These pairs had been collected from the Cairns Region of the Great Barrier Reef and housed at JCU for four years. Adult *A. polyacanthus* and *Am. percula* pairs were maintained under stable, ambient *p*CO_2_ (~490 µatm). Temperatures were increased at a rate of 0.5 °C *per* week until the summer breeding temperature of 29 °C was reached in the first week of November 2015. Adult pairs were provided with half a terracotta pot to act as a shelter and spawning site. Aquaria were checked each morning for the presence of newly laid clutches. Pairs were fed *ad libitum* on commercial fish feed pellets (INVE Aquaculture Nutrition NRD 12/20) once daily outside the breeding season and twice daily during the breeding season (November–May).

*Acanthochromis polyacanthus* juveniles were fed a combination of freshly hatched *Artemia* naupli and weaning fish feed (INVE Aquaculture Nutrition Wean-S 200–400 µm) daily for the first four days post hatch (dph). 5–21 dph they were fed daily on the weaning feed and then switched to a small pellet fish feed (INVE Aquaculture Nutrition NRD 5/8) at 22 dph. Rearing of larval *Am. percula* was performed using methods described by Munday *et al*.^28^. Settled juveniles were fed daily on the weaning fish feed.

### Experimental design

Experiment one was carried out at the aquarium research facility at JCU. For details on the experimental system refer to Supporting Information. Fish were reared at two stable (480 and 1000 µatm) and two cycling (1000 ± 300 and 1000 ± 500 µatm) CO_2_ treatments (Table 1 and Figure S3). The stable 1000 µatm *p*CO_2_ treatment represented the open ocean projection for the end of this century, typically used in many OA experiments^11^. The cycling *p*CO_2_ treatments matched levels that have been observed in some tidal lagoons^38^. Diel *p*CO_2_ fluctuations of between ±50–150 µatm are more typical in other reef areas^40, 41^, however, the magnitude of fluctuations seen in tidal lagoons today may become more common in other reef areas by the year 2100, as a amplification in diel *p*CO_2_ fluctuations is predicted to occur over this time period^5^. Mean values for seawater parameters in experiment one are presented in Table 1.

**Table 1:** Seawater parameters for experiment one. Values are means ± 1 SD for daily average, minimum, maximum and range of pH_T_ and *p*CO_2_.

Parameter	*p*CO_2_ treatment (µatm)			
	480	1000	1000 ± 300	1000 ± 500
Average pH_T_	8.01 ± 0.01	7.75 ± 0.02	7.77 ± 0.08	7.80 ± 0.16
Min. pH_T_	—	—	7.64 ± 0.03	7.57 ± 0.02
Max. pH_T_	—	—	7.89 ± 0.01	8.01 ± 0.01
pH_T_ range	—	—	0.24 ± 0.03	0.44 ± 0.02
Average *p*CO_2_ (µatm)	480 ± 20	990 ± 46	961 ± 195	934 ± 389
Min. *p*CO_2_ (µatm)	—	—	681 ± 21	482 ± 19
Max *p*CO_2_ (µatm)	—	—	1304 ± 102	1591 ± 98
*p*CO_2_ range (µatm)	—	—	623 ± 96	1109 ± 95
TA (µmol kg^−1^)	2570 ± 54	2574 ± 42	2582 ± 43	2583 ± 43
Temperature (°C)	28.7 ± 0.3	28.9 ± 0.3	28.9 ± 0.3	29.0 ± 0.2
Salinity	36.7 ± 0.4	36.7 ± 0.4	36.7 ± 0.4	36.7 ± 0.4

Mean ± 1 SD for total alkalinity (TA), temperature (°C) and salinity over the experiment are also shown.

Experiment two was carried at the National Sea Simulator (SeaSim) facility at the Australian Institute of Marine Science (AIMS) (Cape Cleveland, Australia). Fish were reared at three stable (460, 750 and 1000 µatm) and two cycling (750 ± 300 and 1000 ± 300 µatm) CO_2_ treatments (Table 2 and Figure S4). For details on the experimental system refer to Supplementary Information. Previous experiments indicate that behavioural abnormalities are first evident at around 700 µatm CO_2_, although the magnitude of effect is often not as large as observed at higher CO_2_ levels^27, 28, 31^. Therefore, the inclusion of the 750 and 750 ± 300 µatm CO_2_ treatments enabled us to determine how diel *p*CO_2_ cycles may affect the onset threshold of behavioural abnormalities. Mean values for seawater parameters in experiment two are presented in Table 2.

**Table 2:** Seawater parameters for experiment two. Values are means ± 1 SD for daily average, minimum, maximum and range of *p*CO_2_.

Parameter	*p*CO_2_ treatment (µatm)				
	460	750	750 ± 300	1000	1000 ± 300
Average *p*CO_2_ (µatm)	458 ± 7	748 ± 9	788 ± 203	994 ± 23	1042 ± 256
Min. *p*CO_2_ (µatm)	443 ± 26	721 ± 29	527 ± 27	926 ± 121	667 ± 33
Max *p*CO_2_ (µatm)	477 ± 42	773 ± 28	1025 ± 86	1060 ± 128	1328 ± 78
*p*CO_2_ range (µatm)	49 ± 101	59 ± 112	498 ± 90	130 ± 216	661 ± 94
TA (µmol kg^−1^)	2322 ± 20	2325 ± 23	2326 ± 21	2330 ± 22	2330 ± 23
Temperature (°C)	28.5 ± 0.1	28.5 ± 0.1	28.5 ± 0.1	28.5 ± 0.1	28.5 ± 0.1
Salinity	35.4 ± 0.4	35.4 ± 0.4	35.4 ± 0.4	35.4 ± 0.4	35.4 ± 0.4

Mean ± 1 SD for total alkalinity (TA), temperature (°C) and salinity over the experiment are also shown.

A similar protocol was employed in both experiments. Three offspring clutches were used per species, each from a different parental pair. In experiment one, *A. polyacanthus* and *Am. percula* clutches were transferred to the experimental system and split between *p*CO_2_ treatments in duplicate tanks (12–15 *A. polyacanthus* per tank and 10 *Am. percula* per tank) at 1 and 12 dph respectively. In experiment two, offspring clutches were transferred to the experimental system and split between *p*CO_2_ treatments in duplicate tanks (one tank per line; 15 *A. polyacanthus* per tank and 13–15 *Am. percula* per tank) at 14 and 12 dph respectively. *A. polyacanthus* clutches were transferred at 14 dph in experiment two, compared with 1 dph in experiment one, due to logistical reasons.

Behavioural lateralization trials on *A. polyacanthus* were performed 40–42 dph, which equated to approximately six and four weeks of exposure to *p*CO_2_ treatments in experiments one and two respectively. Predator cue trials on *Am. percula* were performed 18–20 dph, which equated to approximately 1 week of exposure to *p*CO_2_ treatments in both experiments. All behavioural trials were performed between 09:00 and 17:00. Fish were gently transferred to the behavioural arenas using a glass beaker to minimise handling stress. Fish from each *p*CO_2_ treatment were tested at random times throughout the day to account for any possible time of day effects in the fluctuating treatments. Each fish was tested once, being placed in an isolation chamber within their experimental tank after a trial for the rest of the day. Research was carried out under approval of the James Cook University animal ethics committee (permit: A2210) and according to the University’s animal ethics guidelines.

### Behavioural assays

#### Behavioural lateralization trials

Behavioural lateralization (i.e., favoring the left or right side during behavioural activities) is an expression of brain functional asymmetry and a strong determinant of fish behaviour. Lateralized individuals show higher performance in cognitive tasks^64^, schooling behaviour^65^ and escape reactivity^66^. Lateralization in juvenile *A. polyacanthus* was determined using a detour test in a two-way T-maze using methods similar to those described by^31^. The two-way T-maze consisted of an experimental arena (60 cm × 30 cm × 20 cm), with a runway in the middle (25 cm × 2 cm, length × width), and at both ends of the runway (2 cm ahead of the runway) an opaque barrier (12 cm × 12 cm × 1 cm) was positioned perpendicular to the runway. The maze was filled to a depth of 4 cm with the respective treatment water of the fish being tested, being changed after each trial. A single fish was placed at one end of the T-maze and given a 3 min habituation period, during which time it could explore the apparatus. At the end of the habituation period the fish was gently guided into the runway using a plastic rod with the observer standing directly behind the fish (the plastic rod was never placed closer than approximately twice the body length of the fish). At this point to minimise human interference affecting direction turned the observer slowly stepped back from the maze and the fish was allowed to swim to the end of the runway. In instances when a fish did not swim to the end, encouragement was provided by gently moving the plastic rod around at the beginning of the runway. Direction choice was recorded as the first direction turned when the fish exited the runway. Ten consecutive runs were recorded per fish. Twenty fish from each clutch (ten per tank) were tested per CO_2_ treatment. To account for any possible asymmetry in the maze, turns were recorded alternately on the two ends of the runway. Turning preference (i.e. bias in left or right turns) at the population level was assessed using the relative lateralization index (*L*_R_, from −100 to +100, indicating complete preference for left and right turning, respectively) according to the following formula: *L*_R_ = [(Turn to the right − Turn to the left)/(Turn to the right + Turn to the left)] * 100. The strength of lateralization (irrespective of its direction) was also assessed at the individual-level using the absolute lateralization index *L*_*A*_ (ranging from 0 (an individual that turned in equal proportion to the right and to the left) to 100 (an individual that turned right or left on all 10 trials)). Lateralization trials in experiment two were performed with the observer blinded to the experimental treatments.

#### Predator cue trials

The ability to detect and elicit appropriate antipredator behaviour is critical for survival, especially in early life-stages that experience a greater predation threat^67^. The response of juvenile *Am. percula* to a predator cue was tested in a two-channel choice flume using methods similar to those described by^29^. The flume combination was predator cue water *versus* untreated water. Water at the same *p*CO_2_ level from two different sources (9 L buckets) was gravity fed into the choice flume, which was divided down half of its length. A constant flow rate of 100 ml min^−1^ was maintained and monitored using a flow meter and dye test after every water change. Water was changed after each trial. Fish were tested under the mean *p*CO_2_ level of their respective treatments (i.e. fish reared under both 1000 and 1000 ± 300 µatm were tested at 1000 µatm), due to the logistical difficulties involved in manipulating predator cue water pH across a daily cycle. While this resulted in fish from cycling treatments experiencing a change in *p*CO_2_ between experimental and test water, recent work has shown this has no effect on the response of *Am. percula* to a predator cue at far greater changes than experienced in this study^29^. For each trial, a single test fish was placed in the centre of the downstream end of the choice flume and given a 2 min acclimation period. The position of the fish was then recorded every five seconds for a total of 2 min. A rest period of 4 min followed, during which time the water sources were switched to eliminate potential side preferences. The position of the fish was then once again recorded every five seconds for a total of 2 min. Fish were not disturbed during the trial. Temperatures during the trials were kept within 1 °C of the temperature in the rearing tanks. Eight fish from each clutch were tested per *p*CO_2_ treatment (4 per tank). Predator cues were obtained from three common coral-cod, *Cephalopholis miniatus,* as described by ref. 29. Response to predator cue was assessed as the percentage of time spent in the cue water. In experiment one, the control fish from one clutch exhibited no response to the predator cue (i.e. did not avoid the predator cue) and so this clutch was excluded from data analysis.

### Statistical analyses

The effects of *p*CO_2_ treatment on absolute lateralization (*L*_A_), relative lateralization (*L*_R_) and percentage time spent in cue water were tested using mixed-effects logistic regressions^68^. Models for *L*_A_ data from experiment one and predator cue data from experiments one and two were over dispersed and so were re-run using a penalised quasi-likelihood. In all models, parental pair and tank were included as random factors, with tank nested within parental pair. Pairwise comparisons were performed using Tukey’s post hoc tests. To determine if a treatment group demonstrated a turning direction preference Pearson’s Chi-square tests were used, where we expected a 50:50 ratio for left/right turning preference. Finally, differences in the relative frequency distribution of *L*_R_ between treatments were tested using Kolmogorov-smirnov tests. Mixed-effects logistic regressions with and without penalised quasi-likelihood were conducted in R version 3.3.2^69^ using the lme4^70^ and MASS^71^ packages respectively. Pairwise comparisons were conducted using the multcomp^72^ package. Pearson’s Chi-square tests were performed using Minitab 17.

### Data availability

The datasets generated during and analysed during the current study are available from the corresponding author on request or via the Tropical Research Data Hub (doi:10.4225/28/5923bfed71f8d).

## Electronic supplementary material

Supplementary Information
