# Supplementary material for: Diel CO2 cycles reduce severity of behavioural abnormalities in coral reef fish under ocean acidification
Source: Sci Rep. 2017 Aug 31;7:10153. doi: 10.1038/s41598-017-10378-y (PMC5578974; doi:10.1038/s41598-017-10378-y)
Supplement: Supplementary file 1 — Supplementary Information [file 41598_2017_10378_MOESM1_ESM.pdf]

Supplementary Information

Diel CO_2 cycles reduce severity of behavioural abnormalities in coral reef fish under ocean acidification

Michael D. Jarrold[1,2,*], Craig Humphrey[3], Mark I. McCormick[1,2], Philip L. Munday[2]

[1] College of Science and Engineering, James Cook University, Townsville, QLD 4811, Australia.
[2] ARC Centre of Excellence for Coral Reef Studies, James Cook University, Townsville, QLD 4811, Australia.
[3] National Sea Simulator, Australian Institute of Marine Science, PMB 3, Townsville, Queensland 4810, Australia.

*Corresponding author:
Michael D. Jarrold
College of Science and Engineering, James Cook University, Townsville, QLD 4811, Australia.
Email address: michael.jarrold@my.jcu.edu.au

Experimental systems and CO_2 manipulation

Experiment one

The experimental system used at JCU was an 11,000 L re-circulating system. Briefly, the system consisted of a large external 3,700 L sump tank connected to a bio-filter, protein skimmer, UV steriliser and a 1000 L algal bio-remediation tank. The external sump supplied water to four separate 1,600 L re-circulating systems (one *per* pCO$_2$ treatment) made up from one 1000 L sump tank and fifteen 40 L holding tanks, contained within a temperature controlled room. Water was supplied at a rate of approximately 1,600 L *per* day allowing for a complete exchange with the external sump. Holding tanks were supplied with water at a rate of 1 L min^{-1}. Both the internal sumps and holding tanks were aerated with ambient air.

Elevated pCO_2 treatments were achieved by dosing the 1000 L internal sumps with CO_2. This was controlled by solenoid valves (M-Ventil Standard, Aqua Medic, Germany) connected to a pH control system (Aqua Medic AT Control System, Aqua Medic, Germany) with laboratory grade pH electrodes (Neptune Systems, USA). The Aqua Medic AT Control System has a curve function which allowed us to create fluctuating pCO_2 profiles. pH profiles in the fluctuating pCO_2 treatments were recorded every other day using a pH meter (InLab Expert Pro electrode and Seven2Go Pro meter, Mettler Toledo, Switzerland) set to take a reading every 15 min. For the stable pCO_2 treatments pH_{NBS} was measured twice daily using the same model of pH meter. Seawater pH on the total hydrogen ion concentration scale (total scale, pH_T) was measured each week with a spectrophotometer following standard operating procedures[1] using the indicator dye meta/*m*-cresol purple (mCP) (*m*-cresol purple sodium salt 99%, non-purified, Acros Organic). Daily and fluctuating pH_{NBS} measurements were converted to pH_T based on the offset between weekly pH_T and pH_{NBS} measurements. Temperature was recorded daily with a digital thermometer (Comark C26, Norfolk, UK). Salinity readings were taken weekly using a conductivity sensor (HQ15d; Hach, USA). Total alkalinity was also measured weekly using Gran Titration (Metrohm 888 Titrando Titrator Metrohm AG, Switzerland) and certified reference material from Dr. A. G. Dickson (Scripps Institution of Oceanography). All seawater parameters were measured in randomly chosen holding tanks. pCO_2 values were calculated as a function of pH_T, temperature and salinity using CO_2SYS[2] employing constants from[3] refit by[4] and the $KHSO_4$ dissociation constant from[5]. Mean values for each of these seawater parameters are presented in Table 1.

Experiment two

The experimental system used at SeaSim was a flow-through system which comprised of multiple independent lines (duplicate independent lines per pCO_2 treatment). The system used ultra-filtered seawater (0.04 µm), temperature controlled to 28.5°C. Each seawater line supplied three custom made 50 L tanks at the rate of 50 L h^{-1}. The experimental tanks were placed in individual temperature-controlled water baths to ensure temperature stability (± 0.1˚C). Treatments and tank replicates were randomly positioned in the experimental room.

The management of pCO_2 and temperature was achieved through the design and implementation of a custom Model Predictive Control logic running on a micro-PLC (Series S7-1500, Siemens, Australia). The micro-PLC was integrated with the general SeaSim control system, to provided SCADA (Siemens WinCC) accessibility and data archiving. The pCO_2 feedback for each of the replication lines was provided via non-dispersive infrared measurements[1]. Tank water was delivered to the equilibrator (Seasim, AIMS design, custom built) by an in-tank submersible pump (Universal Pump 1260, EHEIM, Deizisau, Germany) where the pCO_2 of the air in the chamber reaches and maintains equilibrium with the pCO_2 of the experimental water. The air was constantly delivered to a NDIR CO_2 analyser (Telaire T6613, Amphenol, Australia) that provided live feedback to the PLC. The CO_2 analysers were calibrated monthly using certified calibration gas mixtures at 0, 600 and 2000 ppm. The control system delivered CO_2 though Gas Mass Flow Controllers (GFC17 series, Aalborg, Orangeburg, USA) according to the profiling schedule designed for the pCO_2 treatment and the feedback signal coming from the experimental tanks. CO_2 was dissolved in the flow-through water by mean of membrane contactors (Membrana Liqui-Cel 2.5x8 Extra-Flow. 3M, USA). Total alkalinity was also measured weekly as described above. Mean values for seawater parameters are presented in Table 2.

Throughout the experiment incoming coastal water had a pCO_2 ranging between 500-550 µatm. Thus, to achieve a control pCO_2 level closer to 460 µatm, membrane contactors (Membrana Liqui-Cel 4x28 Extra-Flow) were used to remove CO_2, using CO_2 – depleted air as sweep gas. This was only possible for the control treatments and consequently the lower pCO_2 levels in the 750 ± 300 µatm treatment matched the pCO_2 of the incoming seawater (500-550 µatm).

Figure S1. Relative lateralization (L_R) for juvenile *Acanthochromis polyacanthus* presented with a T-maze choice chamber in experiment one. Juvenile fish from each pCO_2 treatment (n = 60 per treatment) were allowed to choose to turn left or right for a total of 10 turns. Graphs show L_R with positive and negative values indicating right and left turns, respectively. The extreme values of ∣100∣ indicate fish that turned in the same direction for all 10 turns.

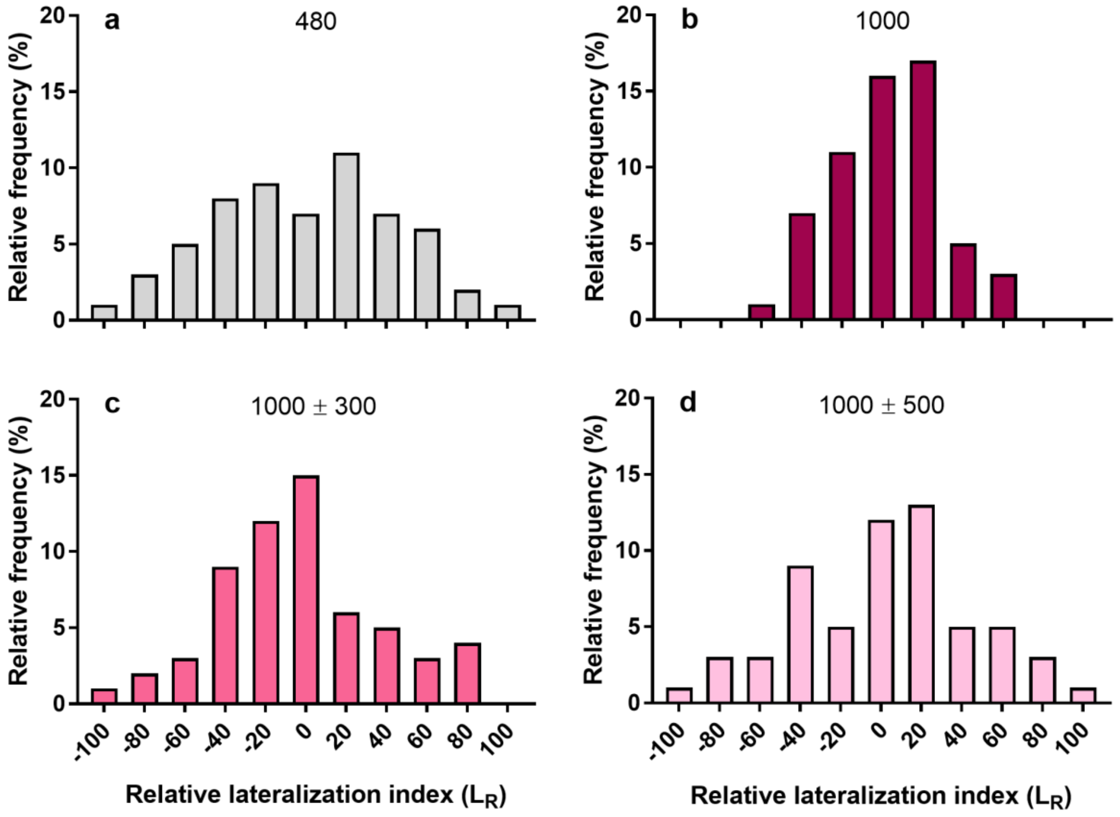

Figure S2. Relative lateralization (L_R) for juvenile *Acanthochromis polyacanthus* presented with a T-maze choice chamber in experiment two. Juvenile fish from each pCO_2 treatment (n = 60 per treatment) were allowed to choose to turn left or right for a total of 10 turns. Graphs show L_R with positive and negative values indicating right and left turns, respectively. The extreme values of $|100|$ indicate fish that turned in the same direction for all 10 turns.

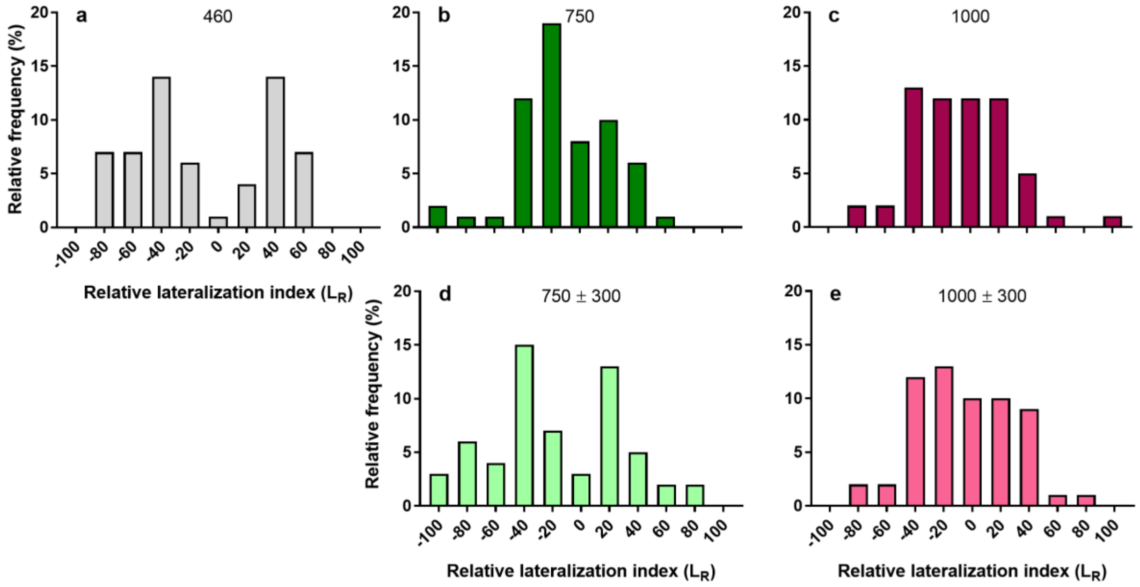

Figure S3. Mean daily pCO_2 profiles for experiment one. Coloured sections are ± 1 SD. Profiles for the stable pCO_2 treatments were based on measurements taken twice per day. In reality some minor daily variation would have likely occurred.

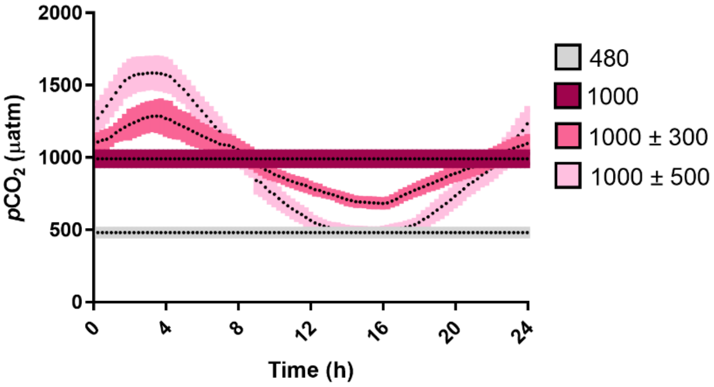

Figure S4. Mean daily (a) stable and (b) cycling pCO_2 profiles for experiment two. Coloured sections are ± 1 SD.

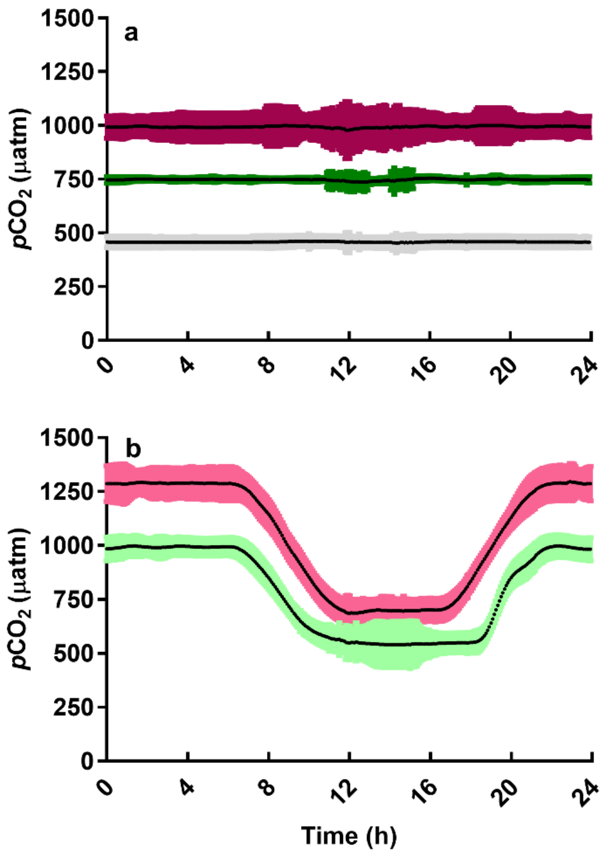

References

1. Dickson, A., Sabine, C. L. & Christian, J. Guide to best practices for ocean CO_2 measurements. PICES special publication 3. IOCCP report No. 8. (2007).

2. Pierrot, D., Lewis, E. & Wallace, D. CO_2SYS DOS program developed for CO_2 system calculations. ORNL/CDIAC-105. Carbon Dioxide Information Analysis Center, Oak Ridge National Laboratory. US Department of Energy, Oak Ridge, Tennessee. (2006).

3. Mehrbach, C., Culberson, C. H., Hawley, J. E. & Pytkowicz, R. M. Measurement of the apparent dissociation constants of carbonic acid in seawater at atmospheric pressure. *Limnol. Oceanogr.* **18,** 897–907 (1973).

4. Dickson, A. G. & Millero, F. J. A comparison of the equilibrium constants for the dissociation of carbonic acid in seawater media. *Deep Sea Res. Part A. Oceanogr. Res. Pap.* **34,** 1733–1743 (1987).

5. Dickson, A. G. Standard potential of the reaction: AgCl(s) + 1/2H_2(g) = Ag(s) + HCl(aq), and and the standard acidity constant of the ion HSO_4^- in synthetic sea water from 273.15 to 318.15 K. *J. Chem. Thermodyn.* **22,** 113–127 (1990).
